# Myeloid Sarcoma: An Unusual Presentation of Acute Promyelocytic Leukemia Causing Spinal Cord Compression

**DOI:** 10.5505/tjh.2012.94809

**Published:** 2012-10-05

**Authors:** Tay Za Kyaw, Jayaranee A.S. Maniam, Ping Chong Bee, Edmund Fui Chin, Veera Sekaran Nadarajan, Hemalatha Shanmugam, Khairul Azmi Abd Kadir

**Affiliations:** 1 University of Malaya, Faculty of Medicine, Department of Pathology, Kuala Lumpur, Malaysia; 2 University of Malaya, Faculty of Medicine, Department of Medicine, Kuala Lumpur, Malaysia; 3 University of Malaya, Faculty of Medicine, Department of Radiology, Kuala Lumpur, Malaysia

**Keywords:** Myeloid sarcoma, Promyelocytic leukemia, Cord compression, Chloroma

## Abstract

Acute promyelocytic leukemia with concurrent myeloid sarcoma is a rare clinical event. Herein we describe a patient that presented with back pain and bilateral leg weakness caused by spinal cord compression due to extramedullary deposition of leukemic cells. Acute promyelocytic leukemia was suspected based on immunophenotypic findings of malignant cells in bone marrow aspirate. The diagnosis was confirmed by the presence of PML-RARα fusion copies. MRI showed multiple hyperintense changes on the vertebral bodies, together with intraspinal masses causing spinal cord compression. The patient immediately underwent radiotherapy, and was treated with all-trans retinoic acid and idarubicin. Reassessment MRI showed complete resolution of all intraspinal masses and the disappearance of most of the bony lesions. Post-treatment bone marrow aspirate showed complete hematological and molecular remission. The motor power of his legs fully recovered from 0/5 to 5/5; however, sensory loss below the T4 level persisted.

## Abstract (tr)

Eş zamanlı miyeloid sarkomlu akut promiyelositik lösemi nadir bir klinik olaydır. Burada ekstramedüller lösemik hücre birikimi nedeniyle spinal kord kompresyonu sonucunda bel ağrısı ve bilateral bacak zayıflığı ile gelen bir hastayı tanımlıyoruz Kemik iliği aspiratında malign hücreler immünofenotipik bulguları temelinde akut promiyelositik lösemiden şüphelenildi. Tanı PML-RARα füzyon kopyalarının varlığıyla doğrulandı. MRG, vertebral cisimlerde çok sayıda hiperintens değişiklik ve beraberinde spinal kord kompresyonuna neden olan intraspinal kitleler gösterdi. Hastaya hemen radyoterapi başlandı ve all-trans retinoik asit ve idarubisin ile tedavi edildi. Tekrar değerlendirmede MRG tüm intraspinal kitlelerin tam olarak geçtiğini ve kemik lezyonlarının çoğunun kaybolduğunu gösterdi. Tedavi sonrası kemik iliği aspiratı tam hematolojik ve moleküler remisyon gösterdi. Bacaklarda motor güç 0/5’ten 5/5’e tam olarak iyileşti ancak T4 seviyesi altındaki duyu kaybı devam etti.

## INTRODUCTION

Myeloid sarcoma (MS) is a rare extramedullary tumorconsisting of immature myeloid cells [1]. MS may developde novo or concurrently with acute myeloid leukemia(AML), myeloproliferative disorders, or myelodysplasticsyndrome. It may proceed or coincide with the occurrenceof AML or may present as the initial manifestationof relapse in a previously treated AML patient in remission[2]. MS may involve any organ system, from the morecommon involvement of the skin, bone, soft tissue of thehead and neck (frequently the orbits) and lymph nodes,to rare cases involving the heart or small intestine [3].The occurrence of MS in an acute promyelocytic leukemia(APL) patient is a rare clinical event. Herein we report apatient with APL that presented with spinal cord compressiondue to underlying MS. 

## CASA PRESENTATION

**Figure 1:**
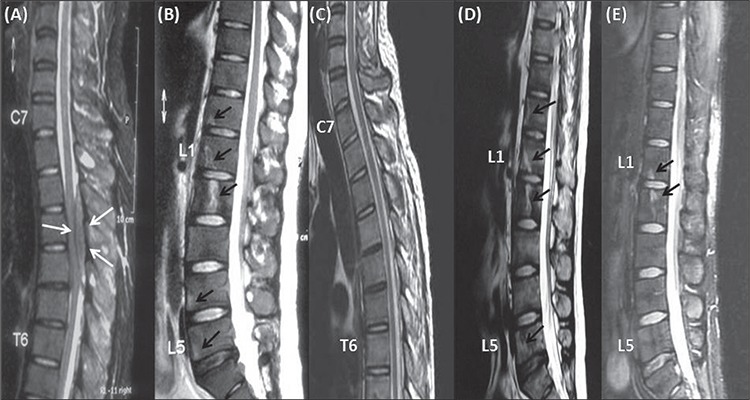
T2 weighted MRI of the patient’s spine. (A+B): Images taken at presentation showing an intraspinal mass (white arrows)located extradurally between T2 and T4 vertebral bodies with multiple hyperintense bony lesions (black arrows) on T12, L1, L2, L4and L5 vertebral bodies. (C+D): Images taken 1 month after treatment showing no evidence of intraspinal mass but the bony lesionson thoracic and lumbar vertebral bodies were persistent (black arrows). (E): Image taken after the third consolidation therapy showinga few residual bony lesions on L1 and L2 vertebral bodies (black arrows).

Written informed consent was obtained from the patient. A 26-year-old male was referred to our hospital due to suspected AML in August 2010. He presented with progressive back pain and bilateral leg weakness, and was unable to walk. Initial neurological examination showed paraparesis with a power of 0/5 in both lower limbs, presence of the Babinski sign, and loss of pain and sensory perception below the T4 level. There were no other abnormal findings on physical examination. Urgent MRI of the spine showed multiple hyperintense bony lesions on the T12, L1, L2, L4, and L5 vertebral bodies, and sacrum. Intraspinal masses located extradurally were also observed between the T2 and T4, and between the T12 and L2 vertebral levels. Theses masses caused anterior displacement and compression of the spinal cord (Figure 1A and B). The patient was immediately referred to a spinal surgeon due to cord compression caused by the intraspinal masses. Radiotherapy was administered immediately at a dose of 20 Gy to 2 regions (T1-T7 and T12-L2) for 5 d. 

**Figure 2:**
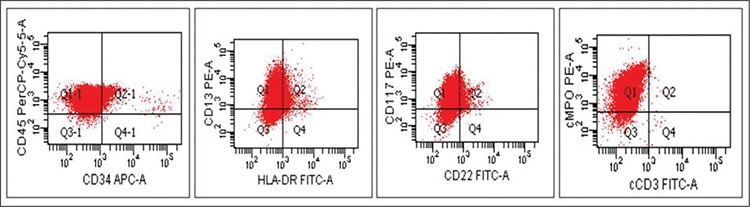
Flow cytometric immunophenotyping of the bone marrow aspirate showing leukemic cells which are CD45 (+),CD13 (+), CD117 (+), cytoplasmic MPO (+), CD34 (-), HLA-DR (-), CD22 (-) and cytoplasmic CD3 (-)

**Figure 3:**
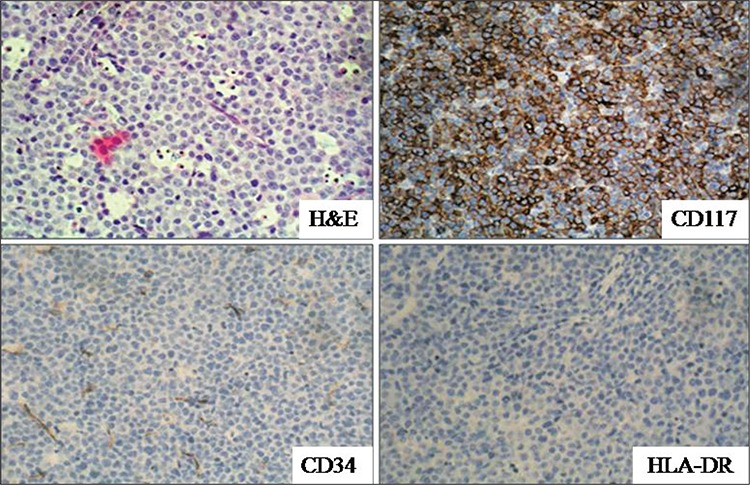
Diffuse infiltration of neoplastic cells in the bone marrow biopsy (H&E, x40). Immunohistochemical analysis of theinfiltrating neoplastic cells showed positive reaction with CD117 (CD117 x40) and negative reaction with CD34 (CD34 x40) andHLA-DR (HLA-DR x40)

Complete blood count upon admission were as follows: hemoglobin: 10.7 g dL^-1^; white blood cell (WBC) count: 2.8 x 109 L^-1^; platelet count: 102 x 109 L^-1^. Occasional blasts were noted in the peripheral blood smear. There wasn’t significant abnormality in the coagulation profile. Cerebrospinal fluid cytology was negative. No conclusive finding was obtained on morphological examination of the bone marrow aspirate, because almost all of the nucleated cells were ruptured. Repeat bone marrow aspirate analysis showed similar findings. Viability of cell suspension in the flow cytometric examination of bone marrow aspirate was 96.6%; this was assessed using 7-amino-actinomycin-D DNA binding dye. Flow cytometric immunophenotyping of bone marrow leukemic cells was suggestive of APL (CD45+, CD13+, CD33+, cytoplasmic MPO+, CD117+, CD64+, CD34-, HLADR-, CD14-, CD11b-, CD19-, CD22-, CD10-, cytoplasmic CD79a-, and cytoplasmic CD3-) (Figure 2). Reverse transcriptasepolymerase chain reaction of the bone marrow sample showed BCR1-type PML-RARα fusion copies, confirming the diagnosis of APL. Bone marrow biopsy also showed diffuse infiltration of the marrow tissue by neoplastic cells (Figure 3). Immunohistochemical staining showed that these infiltrating cells were reactive for CD45, CD117, and MPO. T-cell and B-cell markers (CD3 and CD20), as well as CD34 and HLA-DR were negative (Figure 3). 

Induction therapy consisting of all-trans retinoic acid (ATRA) 45 mg·m^-2^·d^-1^ for 8 weeks, and intravenous idarubicin 12 mg·m^-2^·d^-1^ on d 1, d 3, and d 5 was initiated following molecular confirmation of APL. The treatment course was uneventful. Clinically, motor power of the patient’s lower limbs gradually improved and was restored to 5/5; however, sensory deficits persisted. Repeat MRI of the spine 1 month after completion of radiotherapy showed normal signal intensity of the spinal cord and no evidence of the intraspinal masses, but the bony lesions remained (Figure 1C and D). MRI 17 weeks after the completion of the third cycle of consolidation therapy showed that the bony lesions on the T12, L4, and L5 vertebral bodies, and part of the lesion on the L2 vertebral body disappeared (Figure 1E). No intraspinal masses or new lesions were detected. A biopsy of the residual bony lesions was not performed, as the patient did not consent to the procedure. Bone marrow examination and molecular analysis were performed 3 times—8 weeks after the commencement of ATRA therapy, and 6 weeks and 22 weeks after the third consolidation chemotherapy—and showed complete hematological and molecular remission with undetectable PML-RARα fusion copies. The patient completed 3 cycles of consolidation chemotherapy, and then intrathecal chemotherapy was given for central nervous system (CNS) prophylaxis. At the time this manuscript was prepared the patient was undergoing maintenance therapy.

## DISCUSSION

MS was previously referred to as chloroma due to its characteristic greenish color caused by high myeloperoxidase content [4]. MS at diagnosis or relapse occurs in 3%-8% of AML patients—more frequently in those with myelomonocytic and monocytic morphology (M4 and M5 French-American-British subtypes) [5]. Cytogenetically, MS occurs in association with a variety of chromosomal abnormalities, including MLL gene rearrangement and t(8;21) [6]; the latter more often occurs in childhood and/ or is seen in lesions occurring in the orbit [7]. 

MS in most cases of APL occurs at the time of relapse [6]. In rare cases MS can precede or, as in the presented case, coincide with APL [5,6]. In patients with APL extramedullary disease most commonly occurs in the CNS and ≥10% of hematologic relapses are accompanied by CNS involvement [8]. The occurrence of extramedullary disease in patients with APL has increased since the introduction of ATRA, and 2 possible explanations for this have been considered. The first suggests that there is a direct effect of ATRA on adhesion molecules, resulting in increased infiltrative capability of leukemic cells [9]. The second theory is that the occurrence of MS in relapsed APL patients is a consequence of prolonged survival [10]. A large-scale study that included patients that did and did not receive ATRA therapy failed to demonstrate a correlation between the occurrence of extramedullary disease and ATRA therapy [10]. It has also been suggested that extramedullary tumors may be associated with a high WBC count (>10x10^9^ L^-1^) at presentation, the presence of BCR3-type PML/RARα fusion transcripts, and microgranular morphology. de Bottom et al. reported that 8 of 10 APL patients with extramedullary relapse had elevated WBC counts at initial presentation, and BCR3-type PML/RARα fusion transcripts were present in 6 of the 8 patients [11]. Similar risk factors were not present in the presented patient, who had pancytopenia and BCR1-type PML/RARα fusion copies at presentation. 

Patients with MS can present with various clinical manifestations, depending on the site of involvement. The presented patient presented with the features of spinal cord compression due to lesions in the spine, but tissue biopsy was not performed. Although the bony lesions were persistent in the first repeat MRI, there was complete resolution of the intraspinal masses. Significant improvement was noted in the second MRI, with the disappearance of most of the bony lesions. Post-treatment neurological examination showed complete recovery of the patient’s motor function. The initial MRI findings (concurrent leukemia together with evidence of significant clinical and radiological improvement following treatment), established the diagnosis of MS. The persistence of a few bony lesions in the patient might have been due to residual disease; however, the possibility of radiation-induced changes cannot be completely excluded in the absence of tissue biopsy. Post-treatment bone marrow examination and molecular reassessment, which were performed at 3 time points, showed complete morphological and molecular remission. 

In patients with MS the presence of concurrent AML or APL is easily diagnosed in typical cases in which blasts or abnormal promyelocytes are present in the peripheral blood smear or bone marrow aspirate. In the present patient the morphological findings were not conclusive for the diagnosis of leukemia, as almost all nucleated cells in the bone marrow smears were ruptured. It is likely that the fragile leukemic cells were damaged in the process of making the blood smears. The diagnosis of APL was suspected based on the findings of flow cytometric analysis of viable leukemic cells in the same bone marrow sample. Molecular assessment of the bone marrow aspirate confirmed the presence of PML-RARα transcripts. 

MS and concurrent APL causing spinal cord compression is a rare clinical event, but must be considered in the differential diagnosis in patients that present with neurological symptoms and multiple spinal lesions. Treatment options for MS include surgical decompression, radiotherapy, chemotherapy, and any combination of these treatment methods [12]. The presented patient had significant improvement following local radiotherapy and systemic chemotherapy; however, the presence of residual disease could not be completely excluded, as tissue biopsy was not performed. 

## CONFLICT OF INTEREST STATEMENT

The authors of this paper have no conflicts of interest, including specific financial interests, relationships, and/ or affiliations relevant to the subject matter or materials included.

## References

[1] Neiman RS, Barcos M, Berard C, Bonner H, Mann R, Rydell RE, Bennett JM (1981). Granulocytic sarcoma: Clinicopathologic study of 61 biopsied cases. Cancer.

[2] Pileri SA, Ascani S, Cox MC, Campidelli C, Bacci F, Paccioli M, Piccaluga PP, Agostinelli C, Asioli S, Novero D, Bisceglia M, Ponzoni M, Gentile A, Rinaldi P, Franco V, Vincelli D, Pileri Jr A, Gasbarra R, Falini B, Zinzani PL, Baccarani M (2007). Myeloid sarcoma. Clinico-pathologic, phenotypic and cytogenetic analysis of 92 adult patients. Leukemia.

[3] Antic D, Verstovsek S, Elezovic I, Grujicic D, Gotic M, Bila J, Perunicic M, Jakovic L (2009). Spinal epidural granulocyticsarcoma in non-leukemic patient.. Int J Hematol.

[4] D’Alteroche L, Mor C, Durand V, De Muret A, Benbouker L, Colombat P, Danquechin Dorval E (1999). Gastric granulocytic sarcoma revealed by a massive digestive hemorrhage. Gastroenterol Clin Biol.

[5] Pacilli L, Lo Coco F, Ramadan SM, Giannì L, Pingi A, Remotti D, Majolino I (2010). Promyelocytic Sarcoma of the Spine: A Case Report and Review of the Literature. Adv Hematol.

[6] Campidelli C, Agostinelli C, Stitson R, Pileri SA (2009). Extramedullary Manifestation of Myeloid Disorders. Am J Clin Pathol.

[7] Schwyzer R, Sherman GG, Cohn RJ, Poole JE, Willem P (1998). Granulocytic Sarcoma in Children with Acute Myeloblastic Leukamia and t(8:21). Med Pediatr Oncol.

[8] Evans GD, Grimwade DJ (1999). Extramedullary disease in acute promyelocytic leukemia. Leuk Lymphoma.

[9] Ko BS, Tang JL, Chen YC, Yao M, Wang CH, Shen MC, Tien HF (1999). Extramedullary relapse after all-trans retinoic acid treatment in acute promyelocytic leukemia-the occurrence of retinoic acid syndrome is a risk factor. Leukemia.

[10] Specchia G, Lo Coco F, Vignetti M, Avvisati G, Fazi P, Albano F, Raimondo F, Martino B, Ferrara F, Selleri C, Liso V, Mandelli F (2001). Extramedullary involvement at relapse in acute promyelocytic leukemia patients treated or not with ATRA: A report by the GIMEMA Group. J Clin Oncol.

[11] Botton S, Sanz MA, Chevret S, Dombret H, Martin G, Thomas X, Mediavilla JD, Recher C, Ades L, Quesnel B, Brault P, Fey M, Wandt H, Machover D, Guerci A, Maloisel F, Stoppa AM, Rayon C, Ribera JM, Chomienne C, Degos L, Fenaux P (2006). European APL Group; PETHEMA Group: Extramedullary relapse in acute promyelocytic leukemia treated with all-trans retinoic acid and chemotherapy. Leukemia.

[12] Kalayci M, Sumer M, Yenidunya S, Ozdolap S, Acikgoz B (2005). Spinal granulocytic sarcoma (chloroma) presenting as acute cord compression in a nonleukaemic patient. Neurol India.

